# Structure and function of the alternatively spliced isoforms of the *ecdysone receptor* gene in the Chinese mitten crab, *Eriocheir sinensis*

**DOI:** 10.1038/s41598-017-13474-1

**Published:** 2017-10-11

**Authors:** Xiaowen Chen, Jun Wang, Wucheng Yue, Shu Huang, Jiao Chen, Yipei Chen, Chenghui Wang

**Affiliations:** Key Laboratory of Freshwater Fisheries Germplasm Resources, Ministry of Agriculture, Shanghai Ocean University, Shanghai, 201306 China

## Abstract

Alternative splicing is an essential molecular mechanism that increase the protein diversity of a species to regulate important biological processes. *Ecdysone receptor* (*EcR*), an essential nuclear receptor, is essential in the molting, growth, development, reproduction, and regeneration of crustaceans. In this study, the whole sequence of *EcR* gene from *Eriocheir sinensis* was obtained. The sequence was 45,481 bp in length with 9 exons. Moreover, four alternatively spliced *EcR* isoforms (*Es-EcR-1, Es-EcR-2, Es-EcR-3* and *Es-EcR-*4) were identified. The four isoforms harbored a common A/B domain and a DNA-binding region but different D domains and ligand-binding regions. Three alternative splicing patterns (alternative 5′ splice site, exon skipping, and intron retention) were identified in the four isoforms. Functional studies indicated that the four isoforms have specific functions. *Es-EcR-3* may play essential roles in regulating periodic molting. *Es-EcR-2* may participate in the regulation of ovarian development. Our results indicated that *Es-EcR* has broad regulatory functions in molting and development and established the molecular basis for the investigation of ecdysteroid signaling related pathways in *E. sinensis*.

## Introduction

Ecdysteroids are crucial to the growth, reproduction, development, regeneration and molting of crustaceans^1,2^. The nuclear heterodimeric complex ecdysone receptor (*EcR*)/retinoid X receptor (*RXR*) mediates ecdysteroid activity in crustaceans^3^. Upon activation by ecdysteroids, the heterodimer complex induces the sequential transcription of protein-coding genes that ultimately direct molting, metamorphosis, and growth^4,5^. *EcR* is a member of the nuclear receptor (NR) superfamily and is a ligand-inducible nuclear transcription factor^6^. Like other NR members in crustaceans and insects, *EcR* has a consensus domain architecture that comprises the A/B, C, D and E domains. Among insects, the N-terminal A/B is the least conserved domain and is associated with transcriptional activation. The C domain, also called the DNA-binding domain (DBD), is highly conserved. The D region is a flexible region hinge that mediate nuclear localization and subunit pairing. The E domain contain, also called ligand binding domain (LBD), is moderately conserved and contains a hydrophobic pocket for ligands. Moreover, the E domain is involved in receptor dimerization. In addition to these common domains, a distinct and highly diversified F domain is present in some insects^7–9^.

Alternative splicing is an important mechanism of post-translational modifications, that regulates gene expression and increases gene diversity^10^. A single coding sequence can produce many gene products with different function by alternative splicing, that greatly enrich the biological genetic information^11,12^. Alternative splicing is tightly regulated in a manner that is specific to cell or developmental stage and is a common biological process in human and other organisms^13^. In plants, alternative splicing is pervasive in different developmental stages and environmental conditions^14^. Alternative splicing controls the expression of key sex-determining genes in insects^15^. More than 90% of human genes are alternatively spliced, thus implicating alternative splicing in diverse biological processes^16^. Alternative spliced *EcR* isoforms have been identified and studied in both insects and crustaceans^6,17^. Previous studies have shown that in the *Drosophila melanogaster*, three different functional *EcR* isoforms *EcR-A, EcR-B1* and *EcR-B2* harbor the same DBD and LBD regions, and differ only in their N-terminal regions^18^. Each isoform dominates in different target tissues at different developmental stages^18,19^. Different *EcR* isoforms control cell-specific responses during nervous system remodeling in metamorphosis: for example, *EcR-B* is required for larval molting and neuron remodeling during metamorphosis^20^. Two *EcR* isoforms have different functions during the development of the epidermis and wings of the Tobacco Hornworm, *Manduca sexta* ^21^. Two *EcR* isoforms are expressed in stage- and cell-specific manners during midgut remodeling in the yellow fever mosquito, *Aedes aegypti* ^22^. Alternative *EcR* isoforms have been identified in other insect species with specific functions in different tissues and developmental stages^23–30^.

The crustaceans *EcR* gene has similar functions to insects, but has different numbers and structures of isoforms^6^. Three *EcR* isoforms from the mud crab, *Scylla paramamosain*, were identified with a variable LBD domain^31^. Three isoforms from the water flea *Daphnia magna* exhibit different temporal expression patterns during molting period^32^. An 18-amino-acid insertion/deletion and a 49-amino-acid substitution have been identified in the coding region of *MnEcR* from the freshwater prawn *Macrobrachium nipponense*. These sequence modifications result in splice variants *MnEcR-L1, -L2, -S1* and *-S2* ^33^. Two isoforms have been isolated from the American lobster, *Homarus americanus* ^34^ and four from the blue crab, *Callinectes sapidu* ^35^. However, most studies on crustaceans have only presented the number of alternative *EcR* isoforms. The complete structure of *EcR*, the specific function of each isoform, and the formation of alternative isoforms are less understood in crustaceans. In addition, some of the conserved domains in insects are reportedly different from those of crustaceans^6^. Whether insects and crustacean possess the same evolutionary pattern for *EcR* gene is also unknown.

The Chinese mitten crab, *Eriocheir sinensis*, is an economically important farmed species in East Asia and is an extensively influential species in Europe and Northern America^36,37^. *E. sinensis* presents the representative biological characteristics of crustaceans, such as molting, metamorphosis, regeneration, development, and reproduction^38–41^. Elucidating *EcR* structure and its alternative isoforms will help us decipher the functional roles of *EcR* in *E. sinensis* and expand our knowledge of the crustaceans *EcR*. However, studies that are related to the structure and function of the alternative isoforms in *E. sinensis* are sparse^41^. In the current study, we obtained the whole *EcR* gene sequence, clearly deciphered the *EcR* gene structure, and identified four *EcR* alternative isoforms from *E. sinensis*. To thoroughly understand the biological function of the different isoforms in specific molting stages and tissues, semi-quantitative, quantitative gene expression analyses, and RNA interference experiments were conducted during molting and different tissues of *E. sinensis*.

## Results

### Characterization of the *EcR* gene structure

**Figure 1:**
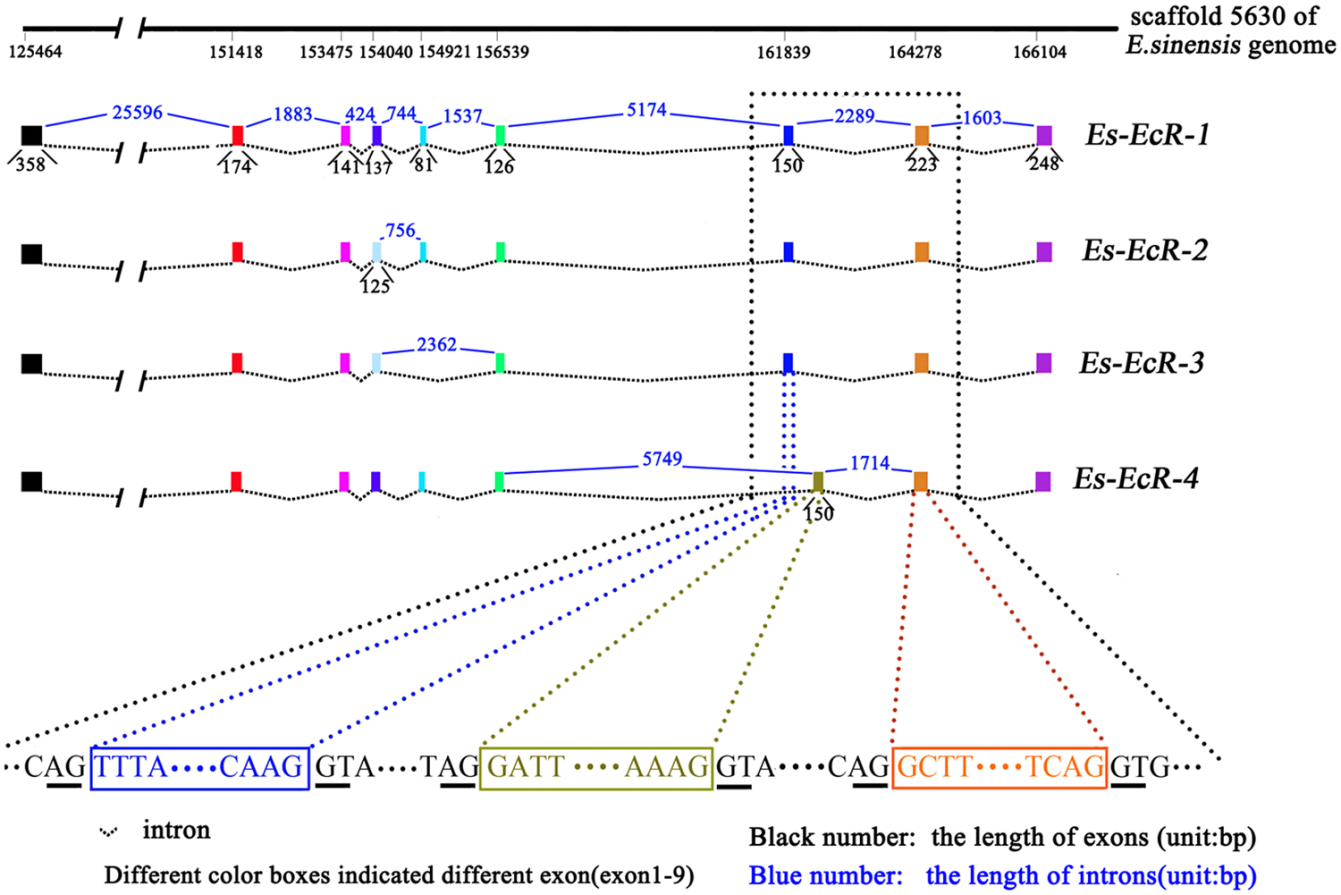
Gene structure of the four *E. sinensis EcR* isoforms. Different colored boxes in the upper figure indicate the different exons (exon 1 to exon 9) of the *EcR* gene. The black underline in the bottom figure denotes the intron boundary. *EcR* gene is located in scaffold5630 of assembled *E. sinensis* genome (http://gigadb.org/dataset/100186)^62^.

The *EcR* gene was 45,481 bp long based on the assembled *E. sinensis* genome from previous study. The gene included 9 exons and 8 introns (accession number KY303919) (Fig. 1, Supplementary Table S2). To verify the accuracy of the assembled *EcR* gene, partial *EcR* DNA sequences were amplified and sequenced by an ABI 3730 sequencer. ORFs were amplified to identify the four alternatively spliced isoforms (*Es*-*EcR-1* to *Es*-*EcR*-*4*). The ORF lengths were: *Es-EcR-1* (1638 bp, accession number KY303915), *Es*-*EcR-2* (1626 bp, accession number KY303916), *Es*-*EcR-3* (1545 bp, accession number KY303917), and *Es-EcR-4* (1638 bp, accession number KY303918) (Fig. 1, Supplementary Fig. S1).

### Alternative splicing patterns of the *EcR* gene

The *E. sinensis EcR* gene had three alternative splicing patterns. The *Es*-*EcR*-2 isoform belonged to the alternative 5′splice site (5′AE). Compared with *Es*-*EcR-1*, a 12 bp sequence on the 3′ ends of exon 4 was absent in *Es*-*EcR*-2 (Fig. 1). *Es*-*EcR-3* lost the entirety of exon 5 and 12 bp from exon 4. This pattern was similar to that in *Es*-*EcR-2*. Thus, *Es*-*EcR*-3 belonged to the 5′AE and exon skipping (SKIP). Although *Es*-*EcR-4* was as long as *Es*-*EcR-1*, but the difference was that *Es*-*EcR-4* lost the whole exon 7 (150 bp in length) compared with *Es*-*EcR-1* to *Es*-*EcR-3*, and a same length intron (150 bp) retained as the exon 7 for *Es*-*EcR-4*. This result indicated that *Es*-*EcR*-4 belonged to exon skipping and intron retention (SKIP and IR) (Fig. 1).

### Functional domain prediction for the four isoforms

**Figure 2:**
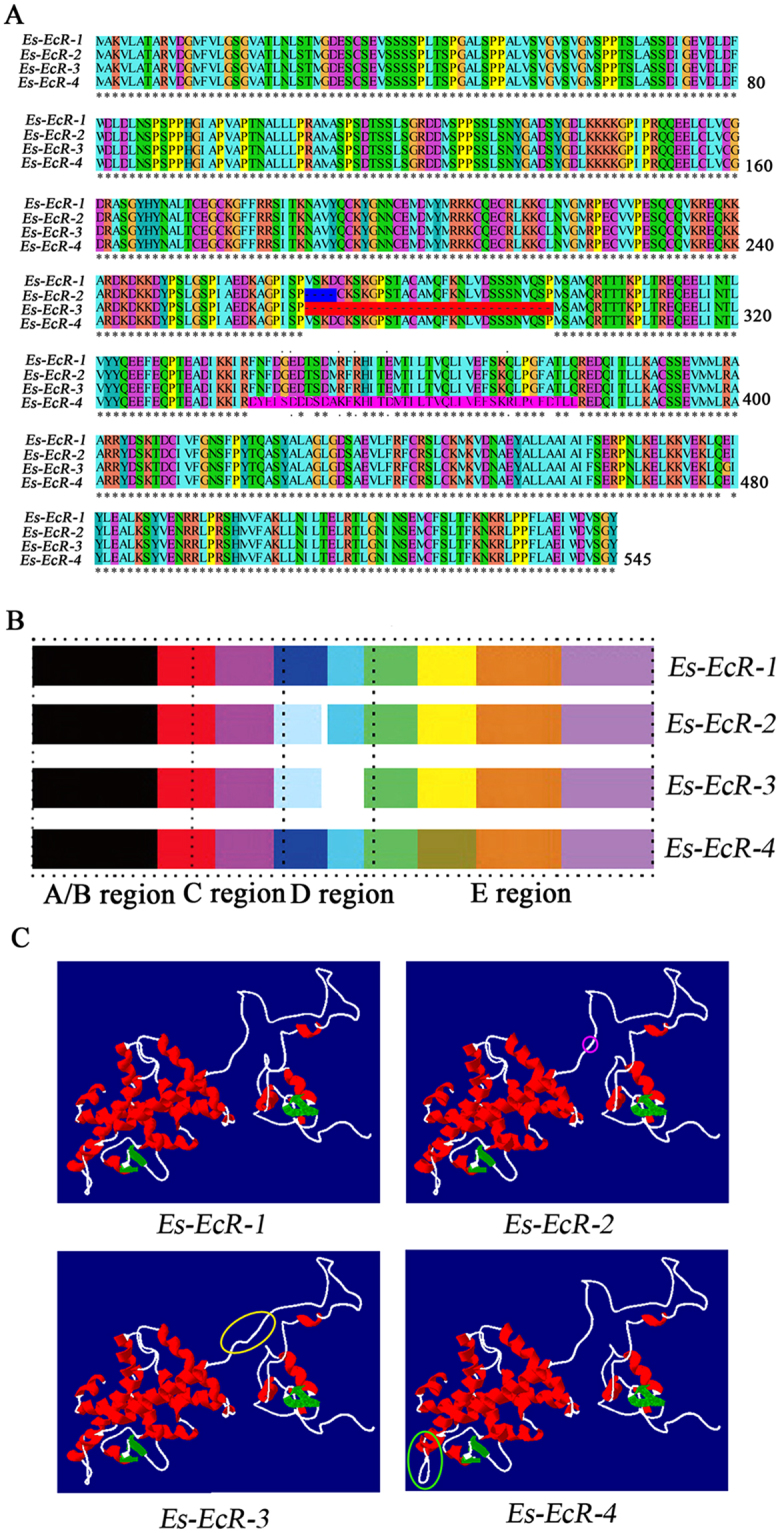
Amino acid sequence alignment, protein domain structure, and predicted protein structure of the four *EcR* isoforms. (**A**) Amino acid sequence alignment of the four *EcR* isoforms. Blue and red boxs indicate the missing sequences of *Es-EcR-2* and *Es-EcR-3*. The purple box denotes the variation in *Es-EcR-4*. (**B**) Pattern diagrams of the domain structures of the four *EcR* isoforms. Variations are identified in the D and E regions. (C region indicates DBD, D region indicates the hinge region between the C and E regions, and E region indicates LBD). (**C**) Predicted protein structure of the four *EcR* isoforms. A purple circle indicates the location of the four missing amino acids in *Es-EcR-2*. A yellow circle signifies the site of missing amino acids; a green circle denotes the location of amino acids variation.

Sequences alignment revealed that all four *EcR* isoforms possessed the four characteristic functional domains of NRs (A/B, C, D, and E domains) (Fig. 2A and B). The A/B and C domains were conserved across the four isoforms, whereas, the lost 12 bp of *Es*-*EcR-2*, as well as the lost 12 bp and the whole exon of *Es*-*EcR-3* were located in the D hinge domain. *Es*-*EcR-4* retained introns in the LBD (E domain). The four alternative isoforms had distinctly different predicted protein structure, which may regulate specific developmental processes or may have different function in *E. sinensis* (Fig. 2C).

### Evolutionary analysis of the *EcR* gene

**Figure 3:**
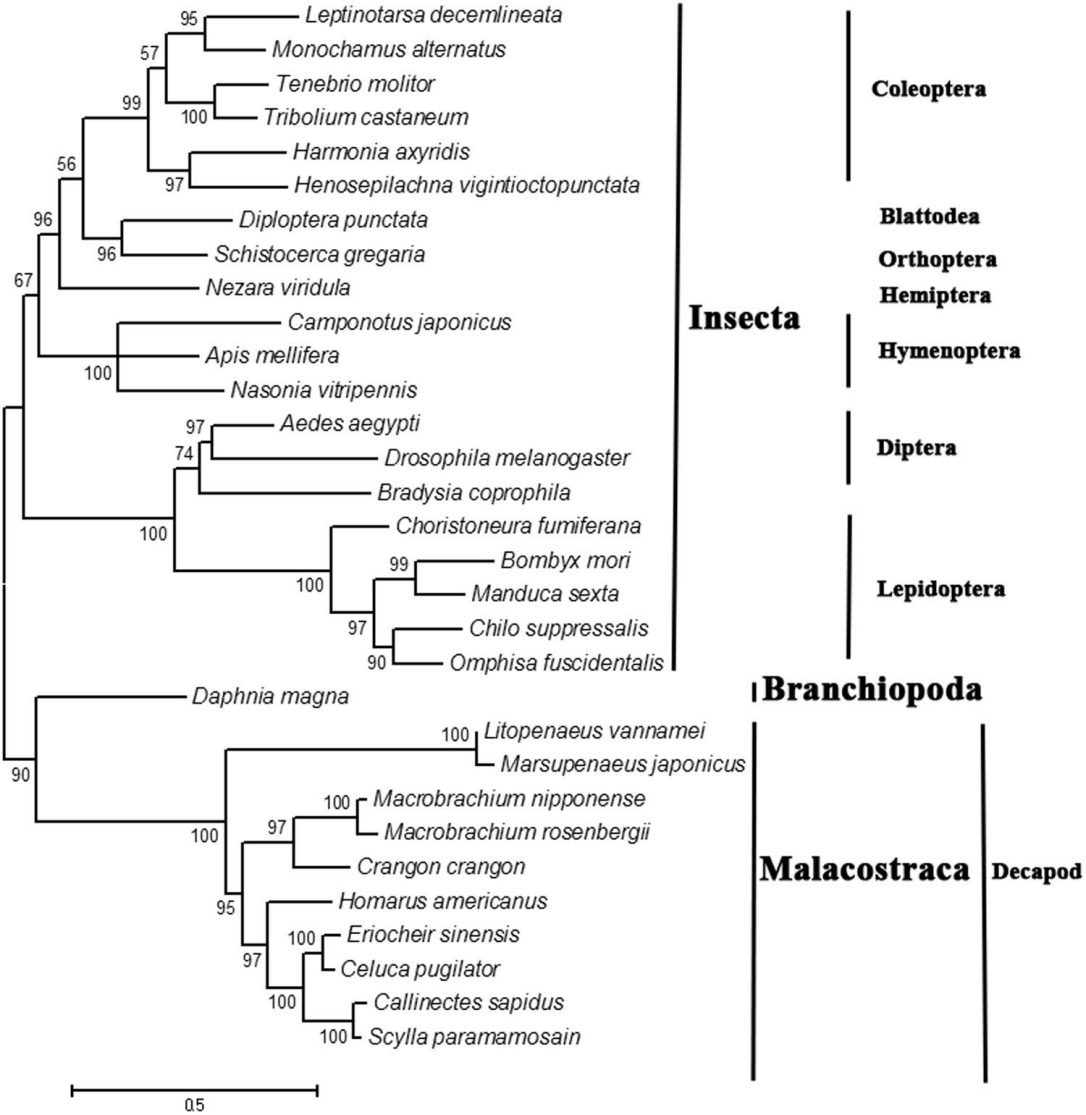
ML phylogenetic tree based on *EcR* sequences. The tree was generated by MEGA 5.0 with 1,000 bootstraps.

The structures of *EcR* genes from 11 crustaceans and 20 insects were observed in this study. The number of alternatively spliced isoforms varied among insects and crustaceans: 10 out of 11 crustaceans harbor more than two isoforms (and most have four isoforms). Moreover, only 3 out of 20 insects had three isoforms and 17 harbored only two isoforms. The variable domains all occurred in the A/B domain of the studied insects. Variable domains occurred frequently in the D and E domains in crustaceans, and 10 out of 11 crustaceans had variable D/E domains (Table 1). We constructed a ML phylogenetic tree with the longest isoforms for each studied species. The tree revealed two independent branches that clearly discriminated between crustaceans and insects (Fig. 3).

**Table 1:** Number of *EcR* isoforms and variable regions in different species.

Species	Taxonomy (subphylum/class)	Number of isoforms	Varied regions	References
*Callinectes sapidus*	Crustacea/Malacostraca	4	D region, E region	35
*Marsupenaeus japonicas*	Crustacea/Malacostraca	4	A/B region	42
			D region,	
			E region	
*Homarus americanus*	Crustacea/Malacostraca	2	E region	34
*Macrobrachium nipponense*	Crustacea/Malacostraca	4	D region, E region	33
*Uca pugilator*	Crustacea/Malacostraca	4	D region, E region	69
*Crangon crangon*	Crustacea/Malacostraca	3	E region	56
*Daphnia magna*	Crustacea/Branchiopoda	3	A/B region	32
Scylla paramamosain	Crustacea/Malacostraca	3	D region, E region	57
*Macrobrachium rosenbergii*	Crustacea/Malacostraca	4	E region	KM886342,
				KM886341
				KM886340,
				KM886339
*Litopenaeus vannamei*	Crustacea/Malacostraca	8	A/B region,	70
			D region,	
			E region	
*Eriocheir sinensis*	Crustacea/Malacostraca	4	D, E region	This study
*Drosophila*	Hexapod/Insecta	3	A/B region	18
*Manduca sexta*	Hexapod/Insecta	2	A/B region	21
*Chilo suppressalis*	Hexapod/Insecta	2	A/B region	23
*Tribolium castaneum*	Hexapod/Insecta	2	A/B region	50
*Choristoneura fumiferana*	Hexapod/Insecta	2	A/B region	37
*Omphisa fuscidentalis*	Hexapod/Insecta	2	A/B region	24
*Camponotus japonicas*	Hexapod/Insecta	2	A/B region	71
*Nasonia vitripennis*	Hexapod/Insecta	2	A/B region	17
*Leptinotarsa decemlineata*	Hexapod/Insecta	2	A/B region	72
*Tenebrio molitor*	Hexapod/Insecta	2	A/B region	73
*Harmonia axyridis*	Hexapod/Insecta	2	A/B region	74
*Epilachna vigintioctopunctata*	Hexapod/Insecta	2	A/B region	74
*Bombyx mori*	Hexapod/Insecta	3	A/B region	75
*A. aegypti*	Hexapod/Insecta	2	A/B region	76
*Diploptera punctata*	Hexapod/Insecta	3	A/B region	27
Monochamus alternatus	Hexapod/Insecta	2	A/B region	77
*Schistocerca gregaria*	Hexapod/Insecta	2	A/B region	78
*Sciara coprophila*	Hexapod/Insecta	2	A/B region	79
*Nezara viridula*	Hexapod/Insecta	2	A/B region	80
*Apis mellifera*	Hexapod/Insecta	2	A/B region	17

### Functional study of splicing isoforms

**Figure 4:**
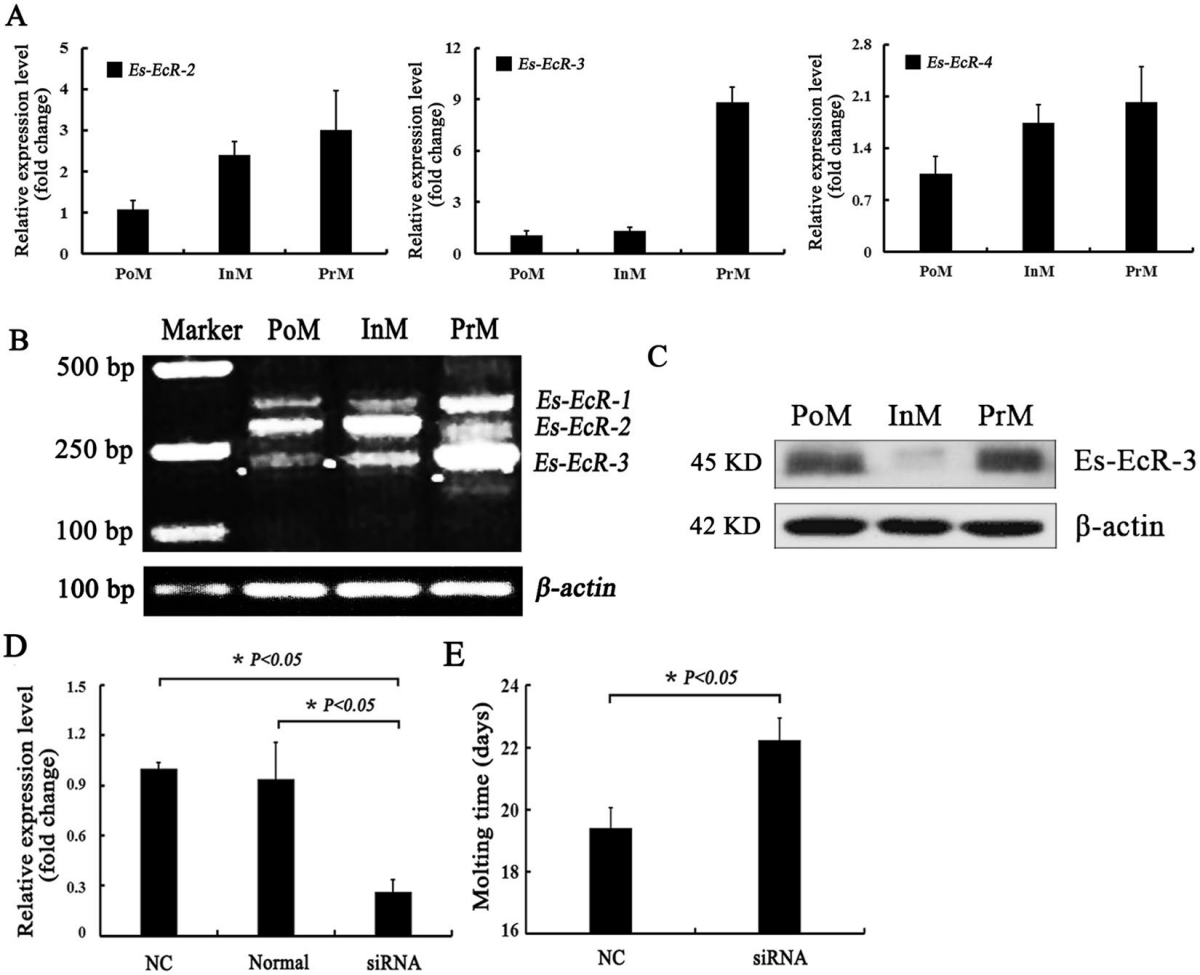
Expression analysis of the four *EcR* isoforms during molting and analysis of siRNA on *Es-EcR-3* isoform. (**A**) qRT-PCR analysis for the relative expression levels of *Es-EcR-2*, *Es-EcR-3*, and *Es-EcR-4*, normalized to the geometric mean of three control genes (*β-actin, S27 and VATB*). Error bars represent standard error. (**B**) Semiquantitative PCR for the expression levels of *Es-EcR-1*, *Es-EcR-2*, and *Es-EcR-3*. (**C**) Western blot analysis for *Es-EcR-3*, and *β-actin* was as reference protein. PoM: postmolt, InM: intermolt, PrM: premolt. (**D**) Relative expression level of *Es-EcR-3* among negative control siRNA (NC), no injection (Normal), and siRNA groups, normalized to the geometric mean of three control genes (*β-actin, S27 and VATB*). (**E**) Lasting days for molting between negative control siRNA (NC) and siRNA groups.

*Es-EcR-2* to *Es-EcR-4* were all expressed during the different molting stages, with the lowest expression level during postmolt stage (PoM) and the highest during premolt stage (PrM) (Fig. 4A). However, no significant difference in the expression of *Es-EcR-2* was identified between intermolt (InM) and PrM stages. *Es-EcR-4* expression levels were not statistically different among molting stages. *Es-EcR-3* expression was nine times higher (fold change) during PrM than during InM (Fig. 4A). Semiquantitative PCR also indicated that *Es-EcR-3* predominated during PrM (Fig. 4B). Western blot results for *Es-EcR-3* were consistent with qRT-PCR and semiquantitative PCR results. *Es-EcR-3* was significantly and highly expressed during PrM (Fig. 4C). The expression level of *Es-EcR-3* was significantly decreased after RNA interference (Fig. 4D) and *in vivo* experiment indicated 3 days was prolonged during molting in siRNA group compared with negative control siRNA group (*P* < 0.05) (Fig. 4E and Supplementary Table S3).

**Figure 5:**
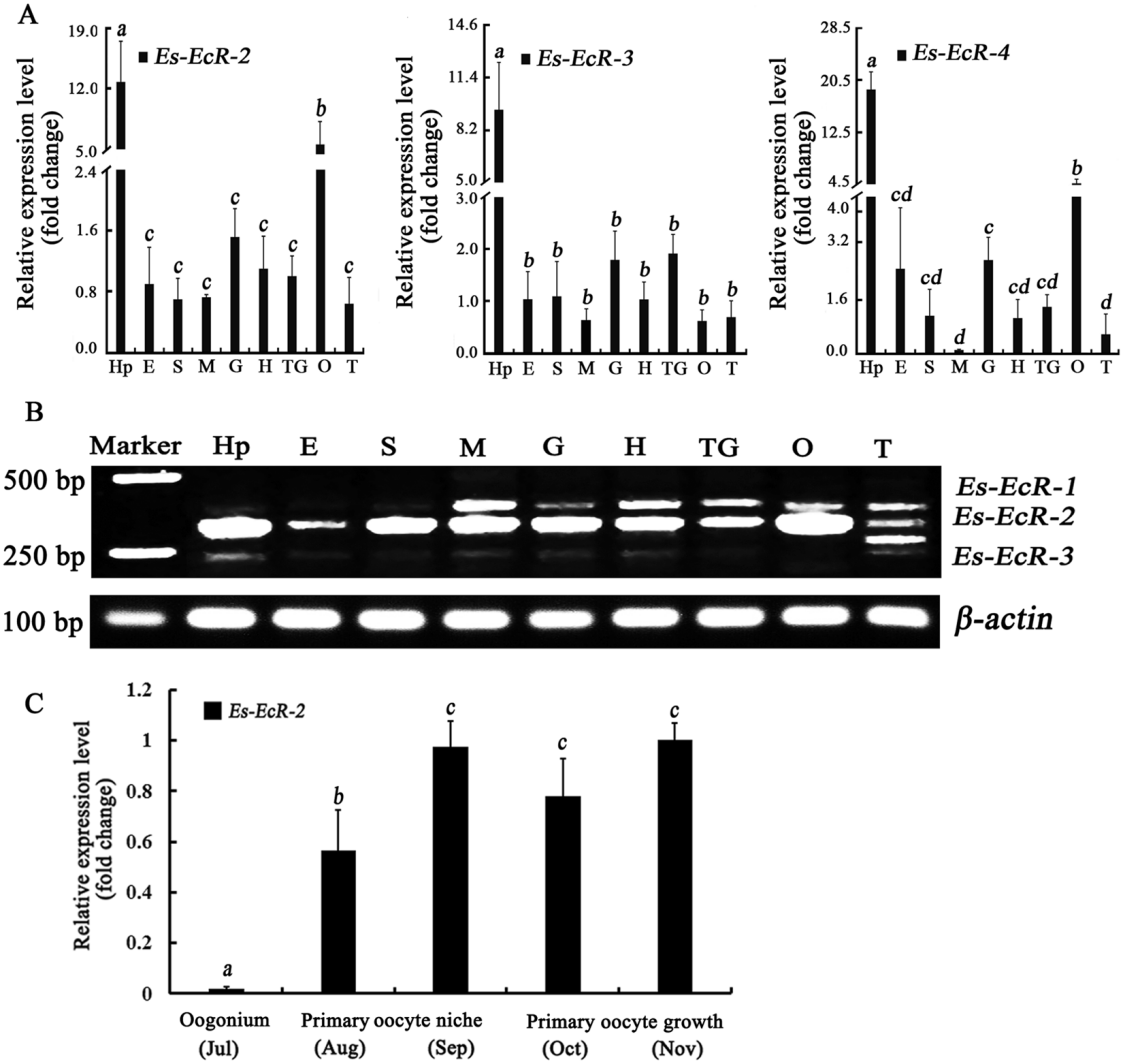
Expression analysis of the four *EcR* isoforms in different tissues. (**A**) qRT-PCR analysis of the relative expression levels of *Es-EcR-2*, *Es-EcR-3*, and *Es-EcR-4* in different tissues, normalized to the geometric mean of 3 control genes (*β-actin, S27 and VATB*). Error bars represent standard error. Groups marked by different *lowercase* letters represented significant difference at *p* < *0.05*. (**B**) Semiquantitative PCR results for the expression levels of *Es-EcR-1*, *Es-EcR-2*, and *Es-EcR-3* in the different tissues. (**C**) qRT-PCR analysis of the relative expression levels of *Es-EcR-2* during different ovary developmental stages. Groups marked by different *lowercase* letters represented significant difference at *p* < *0.05*. Hp: hepatopancreas, E: eyestalks, S: stomach, M: muscle, G: gill, H: heart, TG: thoracic ganglia O: ovary, and T: testis.

qRT-PCR analysis showed that *Es*-*EcR-2, Es-EcR-3* and *Es-EcR*-4 were expressed in all different tissues from adult crabs, with the highest expression in the hepatopancreas. *Es-EcR-2* and *Es-EcR-4* expression levels in the ovary were significantly higher than those in other tissues except in the hepatopancreas. *Es-EcR-3* was expressed at relatively lower levels in the ovary and was not significantly differentially expressed in the ovary and testes (Fig. 5A). Semiquantitative PCR results revealed that the different *EcR* isoforms had different tissue expression patterns. *Es-EcR-1* was expressed at low levels in the hepatopancreas, eyestalks and stomach, but was expressed at relatively high levels in the muscle, heart, thoracic ganglia, and testis. *EsEcR-2* was expressed in all the studied tissues but was highly expressed in the ovary and hepatopancreas (Fig. 5B). The expression level of *Es-EcR-2* was increased gradually during the process of ovarian maturation. The expression level of *Es-EcR-2* was significantly higher in primary oocyte niche and primary oocyte growth stages than oogonium stage (Fig. 5C).

## Discussion

In the present study, we first cloned four alternatively spliced *EcR* isoforms from *E. sinensis*. Based on the assembled draft genome of *E. sinensis*, we identified and evaluated the complete gene structures and preliminary functions of the four *EcR* isoforms. The four isoforms were similar to those in other crustaceans, such as *Callinectes sapidus* and *Marsupenaeus japonicas*. Most crustaceans have more than two *EcR* isoforms (Table 1)^33,35,42^ and most insects harbor two *EcR* isoforms (Table 1)^17,37^. These findings indicate that the crustacean *EcR* gene has a different selection pressures and evolutionary trajectories compared to insects (Table 1, Fig. 3).

As a NR, the insect *EcR* has specific structural characteristics: its DBD and LBD are highly conserved but the A/B domain is highly variable in insects. The conserved DBD and LBD region demonstrates the importance roles of the binding sites, such as *D. melanogaster* ^43–46^, *Tribolium* *castaneum* ^13^, *Apis mellifera* ^17^, and *Chilo suppressalis* ^23^. However, in crustaceans, *EcR* isoforms have a different type of variation in the hinge region (D region) and LBD (E region) (Table 1, Fig. 2). Alternative E region isoforms are unusual among insect NRs, but are common in crustaceans (Table 1). In this study, we confirmed that this pattern exists in *E. sinensis* and may be a specific evolutionary pattern in crustaceans (Figs 1, 2, and Table 1). Our results indicated that the four *EcR* isoforms resulted from the alternative splicing of a single-gene locus and that changeable variant sites likely characterize *EcR* isoforms in crustaceans.

Insects and crustaceans have *EcR* isoforms with specific functions. We found that the four isoforms have fluctuating expression patterns during different molting stages and in the different tissues. Previous studies have reported that the functional specificity of isoforms is linked to their discrete physiological functions^47–50^. Similar to the expression of ecdysone-responsive genes, the expression of *EcR* fluctuates and is essential in the regulation of molting in crustaceans^38^. In our study, *Es-EcR-3* was significantly upregulated during PrM. However, *Es-EcR-2* and *Es-EcR-4* were not differentially expressed during PrM and InM. Furhtermore, knockdown of *Es-EcR-3* caused significantly molting delayed. The findings strongly indicate that *Es-EcR-3* is a vital molting-regulated isoform in *E. sinensis*. Our Western blot results confirmed this hypothesis. As a NR, *EcR* activates gene transcription by binding to specific hormone response elements in the promoters of target genes. The most preferred response element for the *EcR/USP* (*EcR/RXR*) heterodimer is a palindrome element^51,52^. Moreover, the DBD and hinge regions are essential for *EcR/USP* heterodimerization on palindrome elements^53^. The *Es-EcR-3* lost 12 bp and the entirety of exon located in the D hinge domain, which may affect *EcR* and *RXR* heterodimerization and prompt a specific function in molting (Fig. 2B and C). Hinge isoforms are common among NRs. The D domain is important in maintaining the integrity of functional NR structures. Moreover, different hinge isoforms may have different functions^54^. Similar to *Es-EcR-2* and *Es-EcR-3*, which both have variations in the D hinge domain, all hinge isoforms showed specific functions.

Different isoforms are involved in stage- and tissue-specific regulation. *EcR-B1* is predominantly expressed in salivary glands and midgut in *Drosophila* ^55^. *MnEcR* was highly expressed in the hepatopancreas and gills among the ten different examined tissues, and different isoforms predominate in the testis and ovary of the freshwater prawn, *Macrobrachium nipponense* ^33^. *CrcEcR* is expressed in all tissues of the brown shrimp, *Crangon crangon*, and has high expression levels in the ovary, and low in muscle. However, truncated *CrcEcR* isoforms are detected only in the ovary^56^. In our study, *Es-EcR-2* expression levels were higher in the ovary than in other tissues, except hepatopancreas (Fig. 5). *Es-EcR-2* was also predominatly expressed during InM (Fig. 4), which involves energy storage and specific developmental processes^39^. This result signifies that *Es*-*EcR-2* is a developmentally related isoform, and may play essential roles in the growth and the regulation of ovary development^57^. Our qRT-PCR results from different periods of ovary stage also indicated *Es*-*EcR-2* may participate in the course of ovary development. Further studies must be conducted to elucidate the specific functions of *Es-EcR-1* and *Es-EcR-4*.

Several alternative splicing patterns have been reported in previous studies^58^. 5′AE and exon skipping are common splicing patterns. However, an IR splicing pattern in the LBD (E region) domain was identified in this study. Previous studies also showed that this intron retention pattern is also present in the mud crab, *Scylla paramamosain* ^57^. A study on mosquitoes indicated that IR may cause transcript degradation by translation-dependent nonsense-mediated mRNA decay. This process is a potential regulatory mechanism for gene expression in numerous organisms^59^. Therefore, IR in the *Es-EcR-4* gene of *E. sinensis* may be strongly associated with the gene’s specific physiological function. However, we could not fully elucidate this function in the present study.

In conclusion, this paper is the first to report on the four *EcR* isoforms in *E. sinensis*. Our results indicated that the *EcR* genes of crustaceans had different evolutionary trajectories from those of insects. And findings implicate *Es-EcR-3* in the regulation of molting. Moreover, our findings implicate *Es-EcR-2* in the regulation of growth and development, and may participate in ovarian development in *E. sinensis*. Further work must be conducted to interpret the specific function of *Es-EcR-4*.

## Materials and Methods

### Animal and tissue collection

Healthy *E. sinensis* adults were collected from the Nanhui Experiment Station of Shanghai Ocean University (Shanghai, China). Sampling procedures complied with the guidelines of the Institutional Animal Care and Use Committee (IACUC) of Shanghai Ocean University on the care and use of animals for scientific purposes. The experimental protocols were approved by the IACUC of SHOU. Three groups of crabs were sampled under different experimental conditions: (1) Hepatopancraese were sampled at the premolt (PrM), intermolt (InM) and postmolt (PoM) stages^39^, (2) Nine different tissues, including the heart, gill, muscle, hepatopancreas, stomach, thoracic ganglia, ovary, testis, and eyestalk were sampled from each adult crab at the InM stage, (3) Ovary tissues of crabs in July, August, September, October, and November in 2016 were collected, ovary tissues collected from July, August and September, and October and November are considered to be in oogonium stage, primary oocyte niche stage, and primary oocyte growth stage, respectively, according to previous study^60^. Six biological replicate samples were selected per experimental condition. All samples were snap-frozen in liquid nitrogen and subsequently stored at −80 °C before DNA and RNA isolation.

### DNA and RNA isolation and cDNA synthesis

DNA was extracted from the leg muscle of each sample using the saturated sodium chloride method^61^. Total RNA was extracted using an AxyPrep Multisource Total RNA MiniPrep Kit (AxyGen, 09113KD1) and purified with RNase-free DNase I (Tiangen, Beijing, China) to remove DNA contaminates from each sample. The quality of the extracted DNA and RNA was evaluated on 1.0% agarose gels that were stained with ethidium bromide. Reverse-transcription-PCR was performed to synthesize complementary DNA (cDNA) using the PrimeSript™ RT reagent Kit (TaKaRa, Dalian, China) in accordance with the manufacturer’s instructions.

### Sequence amplification of *EcR* gene, and of alternatively spliced isoforms and prediction of protein structure

In order to obtain the *EcR* gene sequence from the recently assembled *E. sinensis*, we used the *EcR* mRNA sequence, which was assembled in our previous study, as “query” to BLAST against the assembled *E. sinensis* genome, then we identified the region and extracted the sequences of *EcR* gene on the assembled *E. sinensis* genome^62^. To amplify and confirm the accuracy of the DNA sequence of *EcR*, primers were designed based on different exons (Table S1). PCR was performed using an Eppendorf Thermal Cycler (Berlin, Germany). The expected PCR products were sequenced with ABI3730 sequencer (Sangon, Shanghai, China). Sequenced sequences were assembled by Cap3 software and the *EcR* gene structure was predicted by Fgenesh software and manually corrected based on the assembled *E. sinensis* genome^63^.

Two alternatively spliced *EcR* isoforms were identified in our previous study^39^. To further confirm and identify novel alternatively spliced *EcR* isoforms, primers were designed to amplify the whole *EcR* open reading frame (ORF) (Supplementary Table S1). cDNA from different tissues and molting stages were mixed and used as a template. PCR was performed using an Eppendorf Thermal Cycler (Berlin, Germany) with a 50 μL reaction mixture that contained 2 U DNA polymerase (Tiangen products, Shanghai, China), 5 μL PCR buffer, 2 μL template cDNA (50 ng/μL), 2 μL dNTPs (0.4 mM), 4 μL primers (0.2 μM each), and 35 μM distilled water. PCR conditions were as follows: 94 °C for 5 min; followed by 30 cycles of 94 °C for 30 s, 58 °C for 30 s, and 72 °C for 2 min; and final extension at 72 °C for 10 min. Expected PCR products were ligated to *PMD19-T* vector and transformed into competent *DH5α* cells. At least 10 positive clones per product were sequenced by ABI3730 sequencer (Sangon, Shanghai, China). Sequences were manually edited by Bioedit software to remove vector sequences and then aligned using CLUSTAL W^64,65^. The alternatively spliced *EcR* isoforms were discovered based on *EcR* gene sequence alignment. The *EcR* gene structure of different isoforms was described by Fancygene software^66^.

The three-dimensional structures of the four isoforms were modelled with the online automated Build Homology Model program at Phyre2 using the crystal structure of the liganded hrxr-alpha/hlxr-beta heterodimer (PDB accession code c4nqaI) as the template (http://www.sbg.bio.ic.ac.uk/phyre2/html/page.cgi?id = index).

### Evolutionary analysis of the *EcR* gene among insects and crustaceans

*EcR* gene sequences from insects and crustaceans were downloaded from NCBI database and previously published papers (Table 1). The number of alternatively spliced isoforms and varied domains were recorded in accordance with published papers. A phylogenetic tree was constructed using Maximum Likelihood (ML) methods with Gamma distributed with Invariant sites (G + I) model implemented in MEGA5 software. The phylogenetic tree was constructed based on available *EcR* sequences from insects and crustaceans with 1,000 bootstraps^67^.

### Quantitative reverse transcription PCR (qRT-PCR) analysis

qRT-PCR was used to quantify the expression level of each *EcR* isoform in different tissues, different molting stages and ovary from different developmental stages. The isoform-specific qPCR primer pairs for *EcR* (*Es*-*EcR*-2, *Es-EcR-3*, and *Es*-*EcR*-4) were designed based on the different sequences of the isoforms (Supplementary Table S1, Supplementary Fig. S1). Given that no workable qRT-PCR primer could differentiate *Es*-*EcR-*1 from other isoforms (*Es*-*EcR*-2 to *Es*-*EcR*-4), *Es*-*EcR*-1 transcript levels could not be determined by qRT-PCR. Semiquantitative PCR was simultaneously conducted for *Es-EcR-1*, *Es-EcR-2*, and *Es-EcR-3*. The isoforms were distinguished based on PCR lengths (Supplementary Table S1). The housekeeping genes, *beta-action* (*β-actin*)*, ribosomal S27 fusion protein* (*s27*), and *vacuolar ATP synthase subunit B* (*vatb*) were used as internal control for qRT-PCR. qRT-PCR was performed with a 25 μL reaction mixture that contained 12.5 μL SYBR Green Premix Ex Taq (Takara, Japan), 1 μL each primer (10 μM), 2 μL diluted cDNA and 8.5 μL dd H_2_O. The PCR procedure used the following program: 95 °C for 30 s, followed by 40 cycles of 95 °C for 5 s, and 60 °C for 30 s. Temperature increased by 0.5 °C/5 s from 60 °C to 95 °C for the melting curve with 30 s elapse time per cycle. The relative expression was estimated using the 2^−∆∆Ct^ method with InM in different molting stage, heart in different tissue, and ovary in November stage as calibration control^68^, the results were present as the fold-change relative to InM, heart, and ovary in November, respectively. Statistical significance (*P* < 0.05) was determined using Student t-tests with SPSS 17.0.

### *Es-EcR-3* polyclonal antibody construction and Western blot analysis

*Es-EcR-3* polyclonal antibody was made according to prokaryotic expression, recombinant protein induction, and polyclonal antibody production procedure. First, the whole *Es-EcR-3* ORF was amplified and inserted into the prokaryotic expression vector, pET-28a; then the expression vector was sent to HuaAn Biotechnology Company (Hangzhou, China) for further recombinant protein induction and polyclonal antibody production. For Western blot analysis, 100 μg total proteins were analyzed in 10% SDS–PAGE (10% acrylamide/bisacrylamide; 350 mM Tris-HCl (pH 8.8); 0.1% SDS; 0.1% ammonium persulfate; TEMED). After electrophoresis, the proteins were transferred onto a polyvinylidene fluoride membrane. The protein blots were blocked with 5% nonfat milk in TBST for 2 h at room temperature (25 °C). Then the membranes were incubated overnight at 4 °C with polyclonal antibodies against *Es-EcR-3* (1:1000, made by Hangzhou HuaAn Biotechnology Company) and a control *β*-actin (1:5000), which was bought from CWBIO Company (Beijing, China). The secondary antibody was anti-rabbit IgG at a dilution of 1:5000. Immunoreactivity was detected with an enhanced chemiluminescence detection kit in accordance with the manufacturer’s instructions (Bio-Rad, Shanghai).

### RNA interference analysis

siRNA was designed based on the specific region of *Es-EcR-3* isoform, and was synthesized by GenePharma Biotech Company (Shanghai, China), which consisted of 21 nt sense and antisense oligonucleotides with 2′ O-Methyl oligo modification. Both siRNA and negative control siRNA were designed (Table S1). In order to test the efficiency of designed siRNA, six crab individuals were injected with siRNA (4 μl, 1 μg/μl), six crab individuals were injected with negative control siRNA (4 μl, 1 μg/ul), respectively, and six crab individuals without injection were used as experimental control. qRT-PCR was conducted on *Es-EcR-3* according to method above (negative control siRNA group was used as calibration control). In order to study the biological function of *Es-EcR-3* on molting, 30 crab individuals (1 ± 0.1 g) were collected immediately after their molting at the same day. Therefore, all the collected 30 crab individuals were at the same molting stage. The 30 crab individuals were randomly divided into two groups, siRNA and negative control siRNA (NC) groups, and were cultured in the same condition. When the crab individuals were at intermolt (InM) stage, 4 μl siRNA (1 μg/μl) and 4 μl negative control siRNA (1 μg/μl) were injected into siRNA and NC groups, respectively, every four days. The molting time was observed and recorded.

## Electronic supplementary material

Supplemental figure

Supplemental table
