# Supplementary material for: Structure and function of the alternatively spliced isoforms of the ecdysone receptor gene in the Chinese mitten crab, Eriocheir sinensis
Source: Sci Rep. 2017 Oct 11;7:12993. doi: 10.1038/s41598-017-13474-1 (PMC5636884; doi:10.1038/s41598-017-13474-1)

Structure and function of the alternatively spliced isoforms of the *ecdysone receptor* gene in the Chinese mitten crab, *Eriocheir sinensis*

Xiaowen Chen[1], Jun Wang[1], Wucheng Yue, Shu Huang, Jiao Chen, Yipei Chen, Chenghui Wang[*]

Supplement

Fig.S1. Nucleotide sequence alignment of four *Eriocheir sinensis EcR* isoforms.

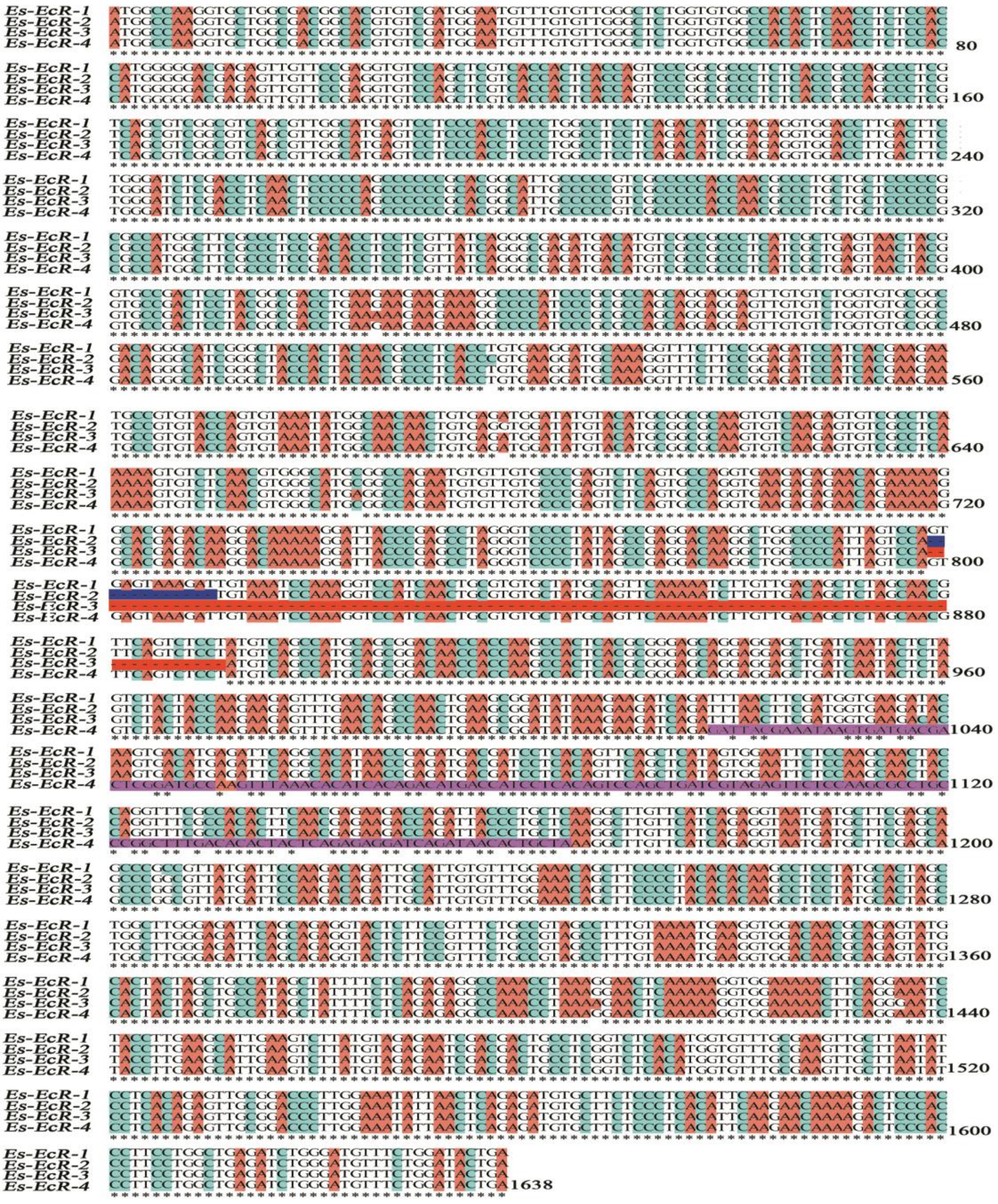

Supplement: Supplementary file 1 — Supplemental figure [file 41598_2017_13474_MOESM1_ESM.pdf]
