# Supplementary material for: Structure and function of the alternatively spliced isoforms of the ecdysone receptor gene in the Chinese mitten crab, Eriocheir sinensis
Source: Sci Rep. 2017 Oct 11;7:12993. doi: 10.1038/s41598-017-13474-1 (PMC5636884; doi:10.1038/s41598-017-13474-1)
Supplement: Supplementary file 2 — Supplemental table [file 41598_2017_13474_MOESM2_ESM.docx]

**Structure and function of the alternatively spliced isoforms of the *ecdysone receptor* gene** **in the Chinese mitten crab, *Eriocheir sinensis***

Xiaowen Chen^1^, Jun Wang^1^, Wucheng Yue, Shu Huang, Jiao Chen, Yipei Chen, Chenghui Wang^*^

**Supplement**

Table S1. Primers used for cloning and expression analysis of *E. sinensis EcR* isoforms.

| Primers | Primers sequences (5’-3’) | Product size (bp) | Targets |
| --- | --- | --- | --- |
| EcR-All | F: TGTTTGTGTTGGGCTCTGGT  R: ATCCCAGATCTCAGCCAGGAA | 1493, 1574,  1586 bp | Identify *EcR* isoform |
| EcR-2 | F: CCCATTAGTCCATGTAAATCCA  R: GCATGGCTGACATAGGAGAC | 106 bp | *Es-EcR-2* qRT-PCR |
| EcR-3 | F: GAGAGAACAGAAAAAGGCACGA  R: ATGGCTGACATTGGACTAATGG | 105 bp | *Es-EcR-3* qRT-PCR |
| EcR-4 | F: AAGTGATGACGACTCGGATG  R: CGCTTGGAGAACTCTACGAT | 87 bp | *Es-EcR-4* qRT-PCR |
| *β*-actin | F: TCATCACCATCGGCAATGA  R: TTGTAAGTGGTCTCGTGGATG | 100 bp | reference gene |
| *S27* | F: GGTCGATGACAATGGCAAGA  R: CCACAGTACTGGCGGTCAAA | 105 bp | reference gene |
| *vatb* | F: TCTTCCTGAACCTGGCCAAT  R: GGACGTGCTTCTCACACTGG | 105 bp | reference gene |
| EcR-semi | F: CCCTATAGCCGAGGACAAGG  R: CATCTCGGTTATGTGCCTGA | 313, 301,  220 bp | Semi-quantitative PCR for distinguish *EcR-1, -2, -3* by length |
| EcR-a | F: AAGAATGCCGTGTACCAGTGT  R: TACTCACTGGACTAATGGGGC | 674 bp | amplify whole intron 3 |
| EcR-b | F: GCCCCATTAGTCCAGTGAGTA  R: TATCCGCTTCAGTTGGCTGTT | 2582 bp | amplify whole intron 4,5 and exon5 |
| EcR-c | F: TGAACAGCCAACTGAAGCGGAT  R: TGAAGTGTGGCGAAACCTGGTA | 5333 bp | amplify whole intron 6 |
| EcR-d | F: GCCACACTTCAACGAGAAGA  R: GCTGTTTCCAAACACAATGC | 2409 bp | amplify whole intron 7 |
| EcR-e | F: GGCGTTATGATTCCAAGACA  R: GCTATGGCAGCTAGTAGTGCA | 175 bp | For identify this sequence in the extron 8 |
| siRNA | Sense: GCCCCAUUAGUCCAAUGUCtt  Antisense: GACAUUGGACUAAUGGGGCtt |  | For *Es-EcR-3* RNA interference |
| Negative control | Sense: UUCUCCGAACGUGUCACGUtt  Antisense: ACGUGACACGUUCGGAGAAtt |  |  |

Table S2. Genomic structure of *EcR* gene

| scaffold504 | Start | End |
| --- | --- | --- |
| exon1 | 125464 | 125821 |
| intron1 | 125822 | 151417 |
| exon2 | 151418 | 151591 |
| intron2 | 151592 | 153474 |
| exon3 | 153475 | 153615 |
| intron3 | 153616 | 154039 |
| exon4 | 154040 | 154176 |
| intron4 | 154177 | 154920 |
| exon5 | 154921 | 155001 |
| intron5 | 155002 | 156538 |
| exon6 | 156539 | 156664 |
| intron6 | 156665 | 161838 |
| exon7 | 161839 | 161988 |
| intron7 | 161989 | 164277 |
| exon8 | 164278 | 164500 |
| intron8 | 164501 | 166103 |
| exon9 | 166104 | 166351 |

Table S3. Detailed information of lasting days for molting between negative control siRNA (NC) and siRNA groups. * indicated crabs died during molting.

|  | 15-18d | 19-22d | 23-26d |
| --- | --- | --- | --- |
| NC | 7 | 7 | 1 |
| SiRNA | 2 | 2+2^＊^ | 9 |
